# Reticulocyte dynamic and hemoglobin variability in hemodialysis patients treated with Darbepoetin alfa and C.E.R.A.: a randomized controlled trial

**DOI:** 10.1186/1471-2369-14-157

**Published:** 2013-07-22

**Authors:** Valentina Forni, Giorgia Bianchi, Adam Ogna, Igor Salvadé, Philippe Vuistiner, Michel Burnier, Luca Gabutti

**Affiliations:** 1 Division of Internal Medicine and Nephrology, Ospedale Regionale di Locarno, via all’Ospedale 1, 6600, Locarno, Switzerland; 2 Division of Nephrology, Lausanne University Hospital, Bugnon 17, 1011, Lausanne, Switzerland; 3 Institute of Social and Preventive Medicine (IUMSP), Lausanne University Hospital, Route de la Corniche 10, 1010, Lausanne, Switzerland

**Keywords:** Erythropoietin stimulating agents, Hemoglobin, Reticulocytes, Variability

## Abstract

**Background:**

In a simulation based on a pharmacokinetic model we demonstrated that increasing the erythropoiesis stimulating agents (ESAs) half-life or shortening their administration interval decreases hemoglobin variability. The benefit of reducing the administration interval was however lessened by the variability induced by more frequent dosage adjustments. The purpose of this study was to analyze the reticulocyte and hemoglobin kinetics and variability under different ESAs and administration intervals in a collective of chronic hemodialysis patients.

**Methods:**

The study was designed as an open-label, randomized, four-period cross-over investigation, including 30 patients under chronic hemodialysis at the regional hospital of Locarno (Switzerland) in February 2010 and lasting 2 years. Four subcutaneous treatment strategies (C.E.R.A. every 4 weeks Q4W and every 2 weeks Q2W, Darbepoetin alfa Q4W and Q2W) were compared with each other. The mean square successive difference of hemoglobin, reticulocyte count and ESAs dose was used to quantify variability. We distinguished a short- and a long-term variability based respectively on the weekly and monthly successive difference.

**Results:**

No difference was found in the mean values of biological parameters (hemoglobin, reticulocytes, and ferritin) between the 4 strategies. ESAs type did not affect hemoglobin and reticulocyte variability, but C.E.R.A induced a more sustained reticulocytes response over time and increased the risk of hemoglobin overshooting (OR 2.7, p = 0.01). Shortening the administration interval lessened the amplitude of reticulocyte count fluctuations but resulted in more frequent ESAs dose adjustments and in amplified reticulocyte and hemoglobin variability. Q2W administration interval was however more favorable in terms of ESAs dose, allowing a 38% C.E.R.A. dose reduction, and no increase of Darbepoetin alfa.

**Conclusions:**

The reticulocyte dynamic was a more sensitive marker of time instability of the hemoglobin response under ESAs therapy. The ESAs administration interval had a greater impact on hemoglobin variability than the ESAs type. The more protracted reticulocyte response induced by C.E.R.A. could explain both, the observed higher risk of overshoot and the significant increase in efficacy when shortening its administration interval.

**Trial registration:**

ClinicalTrials.gov: NCT01666301

## Background

The implementation of erythropoiesis stimulating agents (ESAs) in clinical practice has been one of the most important innovations in the management of anemia in chronic hemodialysis (HD) patients. The ESAs therapy implicates however a non-physiologic stimulation of the erythropoietic process and has been identified as one of the most influential causative factors of hemoglobin variability in HD patients.

Instability over time of the hemoglobin values is a physiological biological event, but is accentuated in HD patients under synthetic drug stimulation of the erythropoiesis [1]. In fact, ESAs therapy induces intermittent peaks of plasmatic erythropoietin (EPO), as compared with the more stable concentration profile of endogenous EPO under the close feed-back loop between erythropoietin concentration and EPO-sensing and producing system acting in physiologic circumstances [1]. Several other aspects of the ESAs therapy may contribute to destabilize the hemoglobin profile over time, such as drug-related factors (pharmacokinetic and bioavailability) and the dose adjustment strategy (doses, dosage frequency) applied by the prescriber [2-5].

The most recent history of ESAs has been signed by the development of long-acting compounds with distinct molecular structure compared with the original epoetin alfa and beta, such as Darbepoetin alfa (half-life 48.8 ± 5.2 hours after subcutaneous (s.c) administration) [6,7] and C.E.R.A. (Continuous erythropoietin receptor activator) (139 ± 20 hours after s.c administration) [8,9], resulting in the recommendation to clinicians to lengthen the administration interval, up to once monthly.

In the absence of evidence about the impact of the half-life and administration interval of ESAs on hemoglobin variability, we first performed a simulation based on a pharmacokinetic model. The task for the nephrologists participating in the study was to achieve and maintain, on a Visual Basic Excel table, the hemoglobin target using 3 different ESAs, with a half-life of 24, 48 and 138 hours, administered weekly or monthly. This study allowed us to demonstrate: (i) the hemoglobin variability decreases with an increase of the ESAs half-life and with a shortening of the administration interval; (ii) the monthly prescription compared to the weekly one is associated with less dosage adjustments, (iii) the number of ESAs posology modifications correlate directly with hemoglobin variability [4].

Aware of the limits of the simulation tool, aiming at verifying our results in the clinical setting and in order to analyze the cellular kinetics of erythopoiesis under different therapeutic strategies, we studied the relationship between two relevant pharmacodynamic response parameters, hemoglobin and reticulocytes, under different ESAs (C.E.R.A and Darbepoetin alfa) and administration intervals (every 2 weeks, Q2W; every 4 weeks, Q4W) in a collective of chronic HD patients.

## Methods

**Figure 1:**
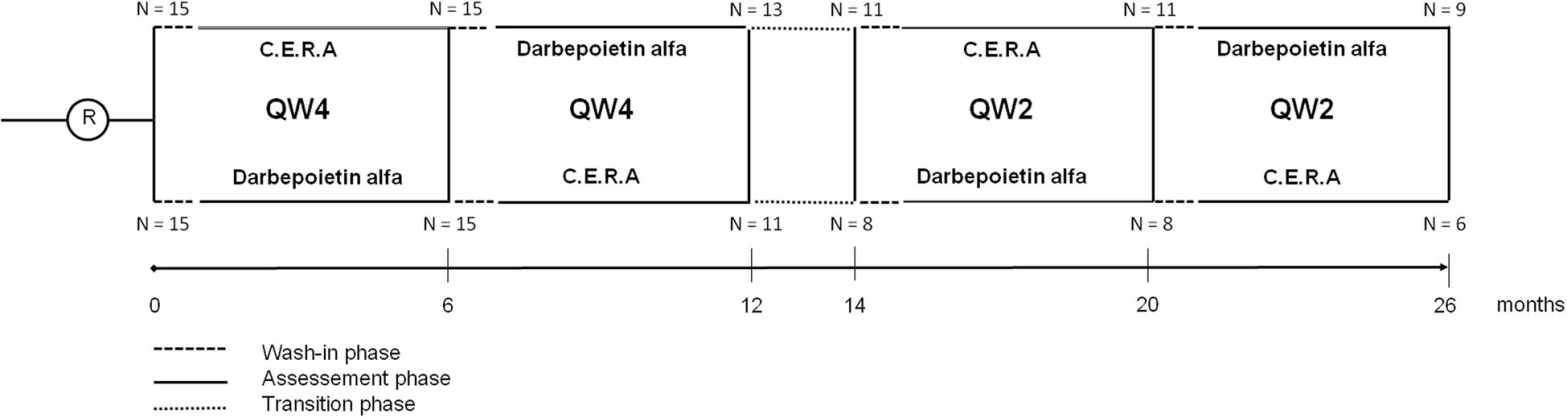
**Study design.** Schema representing the four-period cross-over investigation, composed of 2 periods with ESAs administration scheduled at 4 weeks intervals (Q4W) and 2 periods with ESAs administration scheduled at 2 weeks intervals (Q2W).

The study was designed as an open-label, randomized, four-period cross-over investigation (Figure 1), considering for inclusion all patients under hemodialysis at the regional hospital of Locarno (Switzerland) in February 2010.

We enrolled 31 chronic patients aged 18 years or older, undergoing dialysis 3 times a week for at least 8 weeks before screening, necessitating continuous subcutaneous treatment with weekly Darbepoetin alfa or Erythropoietin beta to maintain hemoglobin (Hb) targets. Exclusion criteria were pregnancy or not respecting the above mentioned conditions. The patients were randomly assigned to subcutaneous C.E.R.A or Darbepoetin alfa, independent of previous ESAs therapy. Patients randomization was conducted by computerized procedure by a co-investigator; the randomization was balanced by groups of six. The randomization codes were communicated to the co-investigators implicated in ESAs prescription and to the dialysis nurses responsible of drug administration. Taking into account the administration of the study drug under supervision, no measure for monitoring compliance was indicated.

Study drug was switched over at the beginning of each treatment period. Each period lasted 6 months; the first month of each period was not considered in the analysis (wash-in phase).

The starting dose of ESA was based on patient’s cumulated dose in the month before randomization according to approved labeling [10].

During the study, doses were adjusted to maintain Hb values within a range of 11.0-13.0 g/dl. Dose adjustments were scheduled at 4 weeks intervals (Q4W) during the first two periods (P1-2) and every 2 weeks (Q2W) during periods three and four (P3-4). P1-2 and P3-4 were intercalated by a 2-month transition phase, during which the drug study was switched but continued to be administrated at Q4W interval (to discriminate between the effect of study drug switch and administration interval change). A protocol directed ESA dose adjustment according to approved labeling [11], based on the most current Hb value measured 1 week previous to the planned administration.

In the Hb target 11.0-13.0 g/dl, dose was continued unchanged. If the rise in Hb was greater than 2 g/dl in four weeks, dose was reduced by 25%. If the Hb exceeded 13.0 g/dl, dose was temporarily withheld until the Hb decreased under 12 g/dl, at which point therapy was reinitiated at 50% lower than the previous dose.

If the increase in Hb was inadequate (less than 1 g/dl), the dose was further increased by 25%.

When switching from Q4W to Q2W administration interval, the monthly ESA total dose was maintained unchanged (conversion factor 1:1), but divided in two administrations. Body weight was not integrated in the ESA dose adjustment protocol.

Of note, Darbepoetin alfa is licensed in Switzerland for one-monthly administration, in contrast to the many other European countries (TIW, Q1W, Q2W).

Supplementary iron carboxymaltose was administered if necessary to stabilize serum ferritin between 100 and 500 μg/l.

Patients were assessed Q1W with hemoglobin values (Hb) and reticulocyte counts (ret), while serum ferritin was measured Q4W.

Hemoglobin variability was the primary outcome while reticulocyte variability, induced reticulocyte response, risk of hemoglobin overshooting and ESAs efficacy comparing 4 to 2 week administration interval were the secondary ones.

The results of the months following a red blood cells transfusion or an overt bleeding episode were not considered in the analysis. Data obtained during patient hospitalization for any reason were similarly suppressed.

The protocol of the study was approved by the local Ethical Committee (Comitato Etico Cantonale della Repubblica e Cantone Ticino). All the patients gave written informed consent prior to enrolling in the study.

### Statistical analysis

To assess the time-variability of both hemoglobin and reticulocytes in Q4W, data were grouped over a four-week period corresponding to the number of weeks following each EPO injection. Linear mixed-effect models were applied on these data with individual random effects. A parabolic function appeared to best fit the data. Adjustments for age and sex as well as for the dose of EPO and iron were made. Reticulocytes were log-transformed to better approach normality. For Q2W, the time between two consecutive injections was too short to allow similar analyses to be performed.

In absence of previous literature permitting an estimation of an expected between-groups difference, no sample size calculation had been driven. Moreover all patients who agreed to participate had been included in the study, so the sample size was the largest we could provide.

The mean square successive difference (MSSD) was used to quantify temporal instability in terms of both variability and temporal dependency over time. The MSSD is the average of the squared difference between successive observations [12]. We distinguished a short-term variability (ST-MSSD) based on the weekly successive difference, from a long-term variability (LT-MSSD) founded on the monthly successive difference.

Both ST-MSSD and LT-MSSD were assessed for hemoglobin, reticulocytes and ESA (only LT-MSSD).

Linear mixed-effect models predicting the effect of ESAs type on the variability (ST-MSSD and LT-MSSD) of hemoglobin were developed, and adjusted for ESAs administration order and ESAs administration interval.

As ST-MSSD and LT-MDDS were right-skewed variables, log-transformed values were used in the analyses to better approach normality.

Statistical analyses were performed using R (version 2.12.0) with the “lme” function from the “nlme” package [13]. Significance level was P < 0.05.

## Results

### Study population

A total of 31 patients were enrolled in the trial and randomized into 2 treatment groups (16 to C.E.R.A and 15 patients to Darbepoetin alfa) at Q4W administration interval. Of these, 30 patients received at least one dose of the study drug, while 1 patient randomized to C.E.R.A died before the first study drug was administrated.

During P1-2, 6 patients prematurely interrupted the assessment because of death (2 in the C.E.R.A., 32 weeks of follow-up lost in P1-2; 2 in the Darbepoetin alfa group, 23 weeks of follow-up lost in P1-2) or renal transplantation (2 in the Darbepoetin alfa group, 16 weeks of follow-up lost in P1-2).

Out of the 24 patients who completed P1-2, 5 patients could not be included in P3-4, because of too low EPO dose, not allowing to be split in 2 half-doses. During P3-4, out of 19 patients (11 of the C.E.R.A and 8 of the Darbepoetin alfa group), 4 patients prematurely interrupted the assessment because of death (1 in the Darbepoetin alfa group, 14 weeks of follow-up lost) or transfer to other hemodialysis centers (2 in the C.E.R.A, 24 weeks of follow-up lost; 1 in the Darbepoetin alfa group, 16 weeks of follow-up lost).

Patient demographic data, clinical characteristics and laboratory results at baseline are presented in Table 1.

**Table 1:** Baseline characteristics of the study participant according to ESAs randomization group during P1

	**C.E.R.A**	**Darbepoetin alfa**	**p-value**
	**(n = 15)**	**(n = 15)**	
**Sex (male), N (%)**	8 (53.3%)	9 (60%)	0.71
**Age, year (mean, SD)**	70.5 (9.7)	69.3 (10.3)	0.73
**Dry weight, kg (mean, SD)**	66.8 (18.5)	77.0 (16.1)	0.12
**Diabetic, %**	53	47	0.71
**Hypertensive, %**	93	93	1.0
**Dialysis duration, hours/week (mean, SD)**	11.5 (1)	11.1 (1)	0.34
**Time on dialysis, years (mean, SD)**	3.1 (2.9)	2.5 (1.9)	0.46
**Hemoglobin, g/dl (mean, SD)**	10.9 (1.3)	11.3 (1.1)	0.37
**Reticulocyte count, cells/μl (mean, SD)**	34147 (12823)	38711 (20368)	0.47
**Ferritin, μg/l (mean, SD)**	357.4 (213.5)	361.8 (127.3)	0.95
**Iron dose, mg/month (mean, SD)**	173.3 (103.3)	186.7 (51.6)	0.66
**ESA dose, μg/month (median, IQR)**	120 (75–158)	100 (64–146)	0.32

*SD* Standard deviation, *IQR* Inter-quartile range.

The most frequent etiologies of chronic kidney disease (CKD) were hypertension (29%), miscellaneous (diabetes and hypertension) (25.8%), diabetes (6.5%), glomerulonephritis (9.7%), interstitial nephritis/pyelonephritis (6.4%) or other/unknown causes (22.6%).

### Efficacy

Data concerning hemoglobin, reticulocyte count, ferritin, cumulated ESAs and iron dose are presented in Table 2 (P1-2, Q4W) and Table 3 (P3-4, Q2W); data comparing P1-2 and P3-4, restricted to the 19 patients who participated in the 2 study phases, are summarized in Table 4. Tables 2, 3, 4, 5 show the combined results of the 2 randomization groups.

**Table 2:** Blood parameter, treatment dose and variability (MSSD) of Period 1–2 (Q4W ESAs administration interval)

	**C.E.R.A**	**Darbepoetin alfa**	**p-value**
	**(n = 30)**	**(n = 30)**	
**Hemoglobin, g/dl (mean, SD)**	11.3 (0.2)	11.2 (0.2)	0.65
**Reticulocyte count, cells/μl (mean, SD)**	69891 (18153)	67047 (18038)	0.55
**Ferritin, μg/l (mean, SD)**	438.7 (190.8)	429.9 (142.7)	0.84
**Cumulated ESA dose, μg (mean, SD)**	788.1 (554.6)	867.9 (721.4)	0.65
**Cumulated iron dose, μg (mean, SD)**	703.7 (325)	781.5 (306.4)	0.37
**ST-MSSD Hb, g**^**2**^**/dl**^**2 **^**(mean, SD)**	0.57 (0.58)	0.52 (0.38)	0.73
**LT-MSSD Hb, g**^**2**^**/dl**^**2 **^**(mean, SD)**	0.62 (0.47)	0.32 (0.36)	0.009
**ST-MSSD Ret, cells**^**2**^**/μl**^**2 **^**(mean, SD)**	26.9x10^8^ (28.2x10^8^)	37.8x10^8^ (36.1x10^8^)	0.20
**LT-MSSD-Ret, cells**^**2**^**/μl**^**2 **^**(mean, SD)**	4.8x10^8^ (5.7x10^8^)	2.5x10^8^ (4.6x10^8^)	0.14

**Table 3:** Blood parameter, treatment dose and variability (MSSD) of Period 3–4 (Q2W ESAs administration interval)

	**C.E.R.A**	**Darbepoetin alfa**	**p-value**
	**(n = 19)**	**(n = 19)**	
**Hemoglobin, g/dl (mean, SD)**	11.6 (0.6)	11.4 (0.6)	0.24
**Reticulocyte count, cells/μl (mean, SD)**	78915 (20625)	72765 (18220)	0.34
**Ferritin, μg/l (mean, SD)**	433.2 (105.3)	478.9 (98.3)	0.18
**Cumulated ESA dose, μg (mean, SD)**	895 (657.7)	865.6 (622.9)	0.89
**Cumulated iron dose, μg (mean, SD)**	712.5 (272.9)	611.1 (286.7)	0.30
**ST-MSSD Hb, g**^**2**^**/dl**^**2 **^**(mean, SD)**	0.52 (0.40)	0.67 (0.45)	0.28
**LT-MSSD Hb, g**^**2**^**/dl**^**2 **^**(mean, SD)**	1.25 (1.10)	1.01 (1.24)	0.54
**ST-MSSD Ret, cells**^**2**^**/μl**^**2 **^**(mean, SD)**	16.5x10^8^ (14.4x10^8^)	20.3x10^8^ (20.5x10^8^)	0.52
**LT-MSSD-Ret , cells**^**2**^**/μl**^**2 **^**(mean, SD)**	12.1x10^8^ (15.5x10^8^)	6.9x10^8^ (9.7 x10^8^)	0.24

**Table 4:** Blood parameter, treatment dose and variability (MSSD) of Period 1–2 vs. Period 3–4, C.E.R.A

**C.E.R.A.**	**Period 1–2**	**Period 3–4**	**p-value**
	**(n = 19)**	**(n = 19)**	
**Hemoglobin, g/dl (mean, SD)**	11.2 (1.2)	11.6 (0.7)	0.16
**Reticulocyte count, cells/μl (mean, SD)**	68367 (17794)	78916 (20626)	0.10
**Ferritin, μg/l (mean, SD)**	444.4 (140.9)	433.1 (105.3)	0.78
**Cumulated ESA dose, μg (mean, SD)**	950.3 (544.5)	895 (657.7)	0.79
**Cumulated iron dose, μg (mean, SD)**	684.2 (285.3)	712.5 (272.9)	0.77
**ST-MSSD Hb, g**^**2**^**/dl**^**2 **^**(mean, SD)**	0.56 (0.67)	0.52 (0.40)	0.83
**LT-MSSD Hb, g**^**2**^**/dl**^**2 **^**(mean, SD)**	0.64 (0.51)	1.25 (1.10)	0.03
**ST-MSSD Ret, cells**^**2**^**/μl**^**2 **^**(mean, SD)**	35.8x10^8^ (29.6x10^8^)	16.5x10^8^ (14.4x10^8^)	0.01
**LT-MSSD Ret, cells**^**2**^**/μl**^**2 **^**(mean, SD)**	5.4x10^8^ (7.3x10^8^)	12.1x10^8^ (15.5x10^8^)	0.10
**LT-MSSD ESA, μg**^**2 **^**(mean, SD)**	10609 (16963)	29043 (12641)	0.25

**Table 5:** Blood parameter, treatment dose and variability (MSSD) of Period 1–2 vs. Period 3–4, Darbepoetin alfa

**Darbepoetin alfa**	**Period 1–2**	**Period 3–4**	**p-value**
	**(n = 19)**	**(n = 19)**	
**Hemoglobin, g/dl (mean, SD)**	11.0 (0.9)	11.4 (0.7)	0.15
**Reticulocyte count, cells/μl (mean, SD)**	67543 (19668)	72766 (18220)	0.41
**Ferritin, μg/l (mean, SD)**	465.9 (156.1)	478.9 (98.3)	0.76
**Cumulated ESA dose, μg (mean, SD)**	1121.6 (744.6)	865.6 (622.9)	0.26
**Cumulated iron dose, μg (mean, SD)**	705.6 (340.4)	611.1 (286.7)	0.37
**ST-MSSD Hb, g**^**2**^**/dl**^**2 **^**(mean, SD)**	0.54 (0.35)	0.67 (0.45)	0.30
**LT-MSSD Hb, g**^**2**^**/dl**^**2 **^**(mean, SD)**	0.28 (0.24)	1.01 (1.24)	0.02
**ST-MSSD Ret, cells**^**2**^**/μl**^**2 **^**(mean, SD)**	48.4x10^8^ (40.3x10^8^)	20.3x10^8^ (20.5x10^8^)	0.01
**LT-MSSD Ret, cells**^**2**^**/μl**^**2 **^**(mean, SD)**	2.0x10^8^ (2.4x10^8^)	6.9x10^8^ (9.7 x10^8^)	0.04
**LT-MSSD ESA, μg**^**2 **^**(mean, SD)**	1701 (2113)	15650 (41055)	0.07

Overall, data of 572 patient-weeks under Darbepoetin alfa and 571 patient-weeks under C.E.R.A in P1-2, respectively 353 patient-weeks under Darbepoetin alfa and 359 patient-weeks under C.E.R.A in P3-4, were analyzed.

**Figure 2:**
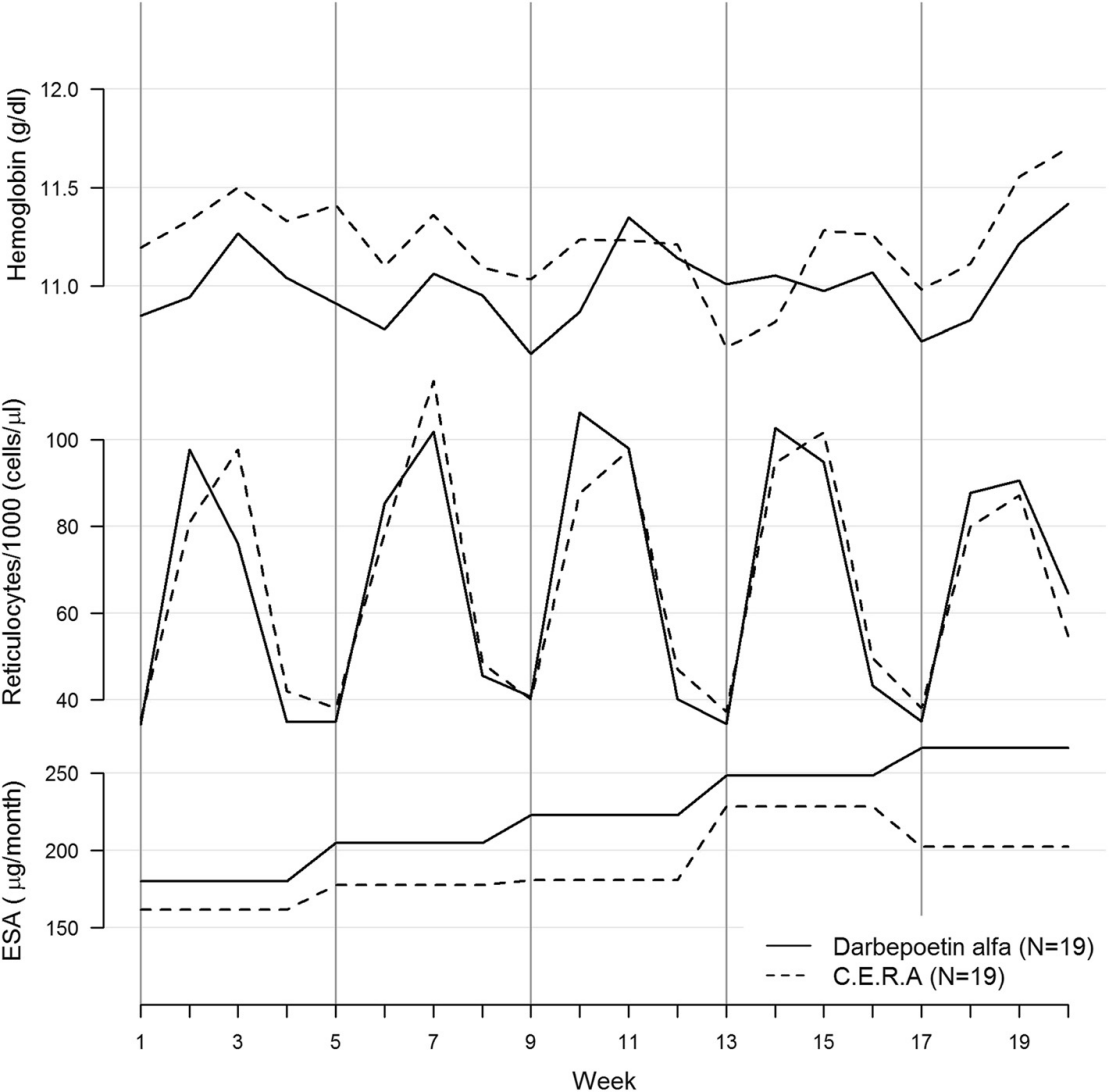
**Hemoglobin and reticulocyte time course, Q4W administration interval.** Hb, ret and ESA dose as a function of time at 4 weeks ESAs-administration interval; C.E.R.A. black dash line, Darbepoietin alfa black solid line. Vertical lines correspond to ESA administration.

**With Q4W ESAs administration (P1-2)** (Figure 2), hemoglobin response to subcutaneous ESA was characterized by an oscillatory movement over 1 month, reaching the maximal level about 2½ weeks after injection. There were no significant interactions between ESAs type and time (p = 0.7) nor time^2^ (p = 0.8), suggesting any significant time course difference of hemoglobin response between ESAs. The hemoglobin level was slightly higher in participants under C.E.R.A than in those under Darbepoetin alfa, but the difference was not significant (p = 0.2). Similarly, the reticulocyte response followed an oscillatory cyclic movement over 1 month with both ESAs. The monthly oscillatory time course differed between C.E.R.A and Darbepoetin alfa (the interaction terms were clearly significant: p < 0.001 for time and p < 0.001 for time^2^). In participants under Darbepoetin alfa, the reticulocyte level reached its maximum within the first days after injection but it then decreased almost linearly, whereas under C.E.R.A, the maximum level was slightly lower, but it remained nearly constant up to two weeks after the injection (secondary outcome). We found a significant correlation between hemoglobin and reticulocyte count (p < 0.001) after adjusting for time, ESAs type, baseline Hb, ESAs-dose and cumulated dose, iron dose and cumulated iron.

**Figure 3:**
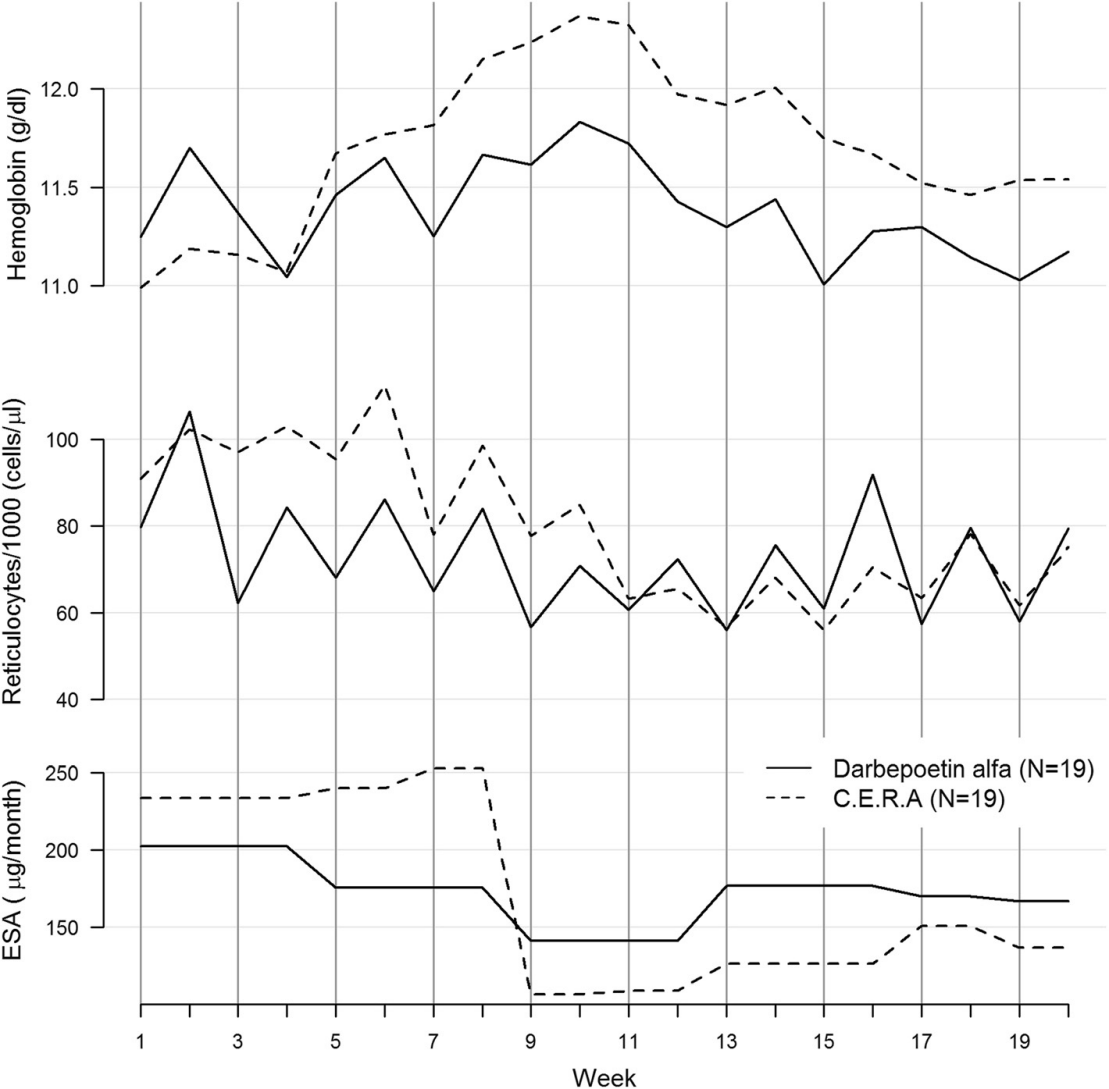
**Hemoglobin and reticulocyte time course, Q2W administration interval.** Hb, ret and ESA dose as a function of time at 4 weeks ESAs-administration interval; C.E.R.A. black dash line, Darbepoietin alfa black solid line. Vertical lines correspond to ESA administration.

**With Q2W ESAs administration (P3-4)** (Figure 3), the hemoglobin time course became more irregular. We observed, indeed, a loss of the systemic monthly cyclic behavior, characterizing P1-2.

The reticulocyte response preserved a cyclic oscillatory course over 1 month. Unlike the monophasic movement observed in P1-2, the oscillation became biphasic in P3-4, reaching its peak within the first week after ESAs injection and returning to the baseline within 2 weeks.

Data concerning short-term (week-to-week) variability (ST-MSSD) and long-term (month-to-month) variability (LT-MSSD) are summarized in Tables 2, 3, 4, 5.

For hemoglobin, no significant difference was found in ST-MSSD, when modifying ESAs or administration interval. However, a significant hemoglobin LT-MSSD increase was found when shortening the administration interval from Q4W to Q2W, both for C.E.R.A (p = 0.03) and Darbepoetin alfa (p = 0.02) (primary outcome). Reticulocyte LT-MSSD was found to predict hemoglobin LT-MSSD (p < 0.0001), after adjusting for ESA type, administration order and interval.

Reticulocyte LT-MSSD increased when shortening the administration interval with both ESAs. The difference was significant in the Darbepoetin alfa group (p = 0.04), but didn’t reach significance in the C.E.R.A. group (p = 0.1).

Reticulocyte ST-MSSD decreased significantly when shortening the administration interval from Q4W to Q2W, both for C.E.R.A (p = 0.01) and Darbepoetin alpha (p = 0.01).

**Figure 4:**
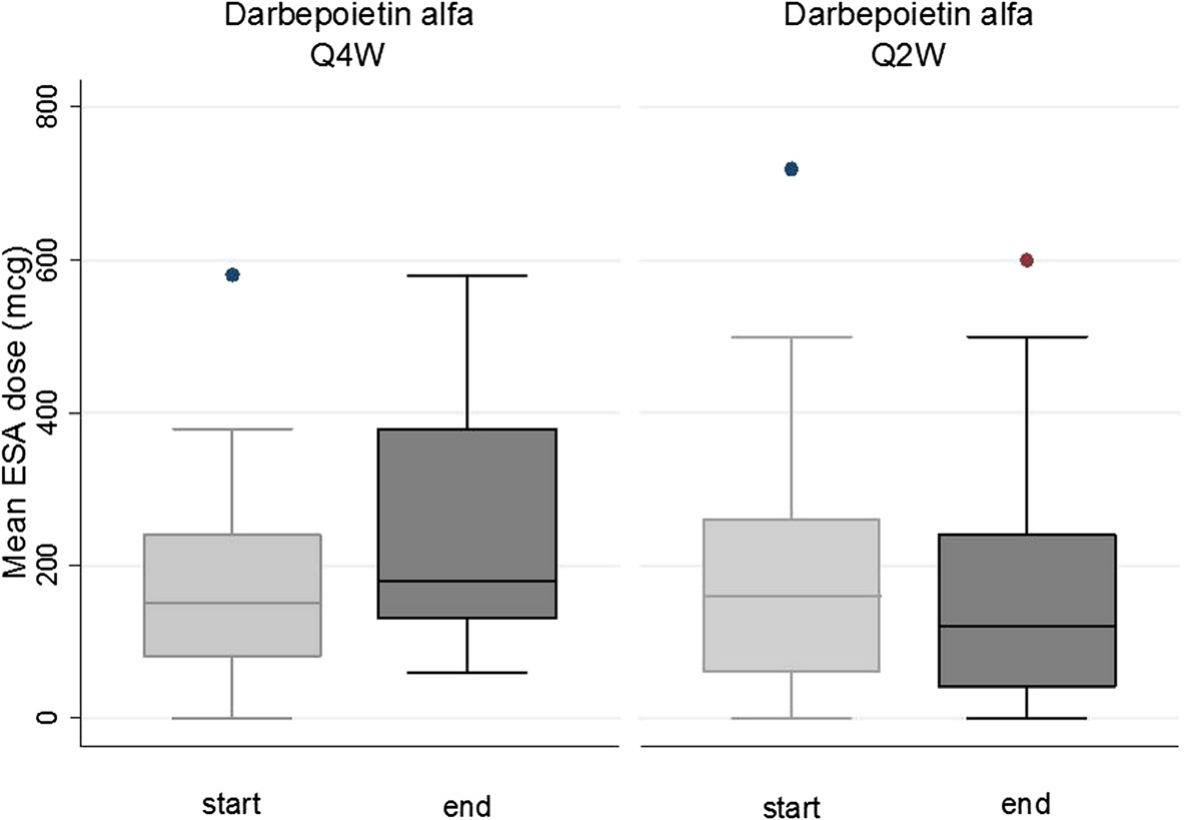
**Start- and end- monthly ESA dose, Darbepoetin alfa.** Starting and end-monthly dose of Darbepoietin alfa at 4 weeks (Q4W) and 2 weeks (Q2W ) administration intervals. Boxes represent 25th and 75th percentiles, whiskers 5th and 95th percentiles. N = 19.

**Figure 5:**
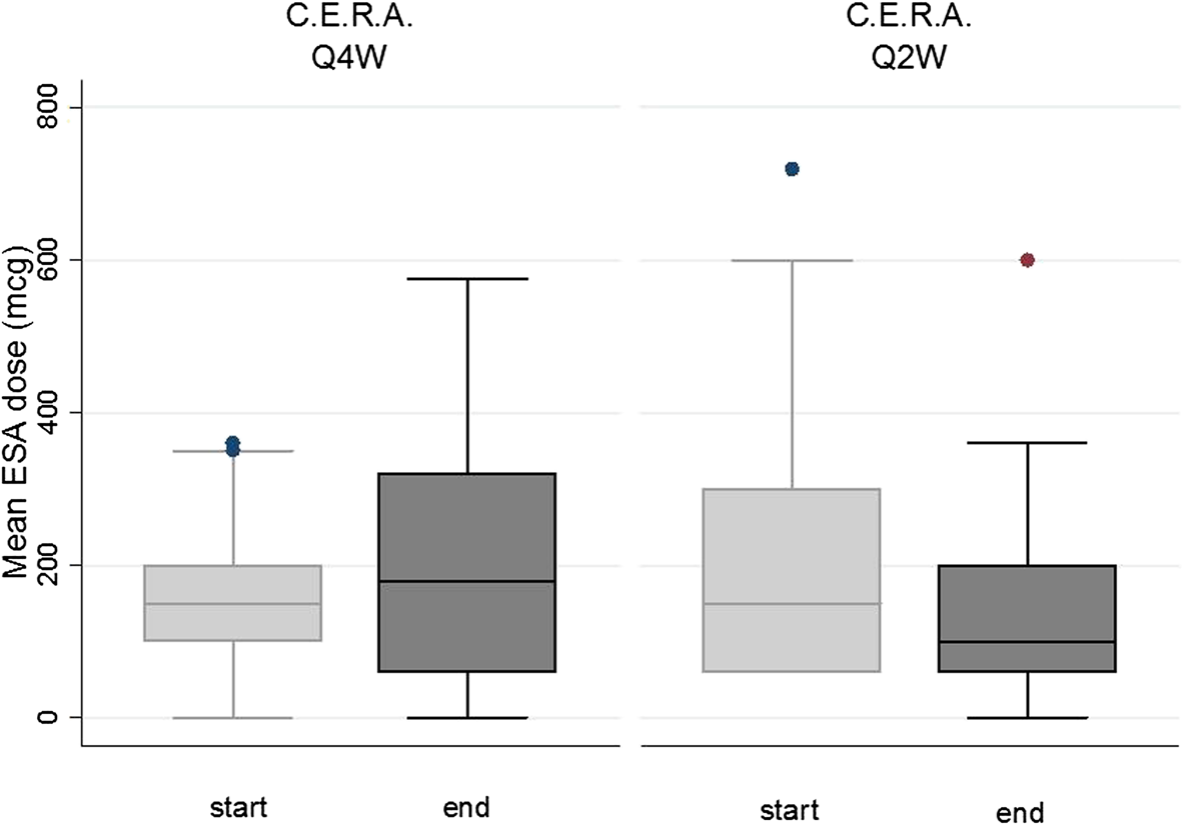
**Start- and end- monthly ESA dose, C.E.R.A.** Starting and end-monthly dose of C.E.R.A at 4 weeks (Q4W) and 2 weeks (Q2W) administration intervals. Boxes represent 25th and 75th percentiles, whiskers 5th and 95th percentiles. N = 19.

Concerning ESAs, when analyzing the start and final dose utilized in both study phases, we observed that: Darbepoetin alfa monthly dose significantly increased from mean (sd) 180 (145) to 266 (172) μg over P1-2 (p = 0.002), without significant change over P3-4, from mean (sd) 202 (185) to 170 (168) μg (p = 0.3) (Figure 4); C.E.R.A monthly dose was unchanged over P1-2, from mean (sd) 161 (106) to 202 (167) μg (p = 0.3) , but significantly decreased over period P3-4, from 244 (222) to 151 (154) μg (p = 0.01) (Figure 5) (secondary outcome). After adjustment for confounding factors (ESAs administration interval, cumulated ESAs dose prior to the event, cumulated iron dose), the risk of Hb overshooting (defined as Hb > 12.0 g/dl) was found to be predicted from ESA type, with a higher risk for C.E.R.A. (OR 2.7, p = 0.01) (secondary outcome). ESAs type and administration interval were not found to predict Hb values < 10 g/dl, which were correlated with cumulated ESA dose (p = 0.02).

9 patients necessitated red blood cells transfusion during the study, in 2 cases for overt bleeding, in 6 cases after a surgical intervention and the remaining in the course of an infectious disease. Because of transfusion, data of 36 weeks were not considered in analyses. 33 hospitalizations occurred during the study, generating a loss of 52 weeks of follow-up.

## Discussion

The current study was designed to examine the influence of two long half-life erythropoiesis stimulating agents (ESAs) administered subcutaneously at different intervals on the cellular kinetics of erythropoiesis under conditions of standard clinical practice in hemodialysis patients and to verify the findings of a simulation based on a pharmacokinetic model suggesting a correlation between hemoglobin variability and both half-life and administration interval [4].

Hemoglobin in dialysis patients is an instable parameter, influenced by several measure-related (analysis bias), patients-related (hydration [14], inflammation [15,16], nutrition [17,18], blood loss, hospitalization [5]) and treatment-related factors (dialysis adequacy [19], administration of intravenous iron, correction of iron deficiency and ESAs therapy [5]).

Data comparing the influence of different ESAs on hemoglobin variability are scarce and contrasting [5,20-22]. The primordial challenge in this research field is how to define and assess correctly “variability”. Several statistical indices (within-person variance or standard deviation, autocorrelation …) have been used and several definitions have been formulated. The most common one is to define a non-physiological “cycle”, an “oscillation of Hb >1.5 g/dL over >8 weeks, during which Hb levels increased or decreased and then reverse to the initial trajectory” [5]. The limits of most of these indices and definitions, are that they do not consider that hemoglobin acts as a non-stationary time series, in which a systematic increase or decrease in overall level of response occurs over time (temporal dependency).

Unlike the standard deviation and the autocorrelation, measures based on successive change allow quantifying temporal instability in terms of both variability and temporal dependency over time. An example is the mean square successive differences (MSSD), which implies the average of the squared difference between successive observations. Further, the MSSD allows to distinct short-term (e.g. week-to-week) from long-term instability, producing fluctuations across a long-term duration (e.g. month-to-month). MSSD could be a more precise estimator of hemoglobin instability over time.

A second important limit of the studies about hemoglobin variability under ESAs is that they ignore the effect of ESAs on cellular production, namely the ESAs induced reticulocyte response. If and how reticulocytes are implicated in the phenomenon of hemoglobin variability is actually not known.

With the current four-period cross-over study, we analyzed the influence of 2 ESAs (C.E.R.A. and Darbepoietin alfa) and 2 administration intervals (Q4W, Q2W) on hemoglobin and reticulocyte response. Therefore, 4 treatment strategies (C.E.R.A./Q4W, C.E.R.A./Q2W, Darbepoetin alfa/Q4W, Darbepoetin alfa/Q2W) were compared with each other.

No difference was found in the mean values of biological parameters (hemoglobin, reticulocytes, and ferritin) between the 4 strategies. However, Q2W administration interval seemed to be more favorable in terms of ESAs dose, allowing over the 6 observation months a 38% C.E.R.A. dose reduction, and no increase of Darbepoetin alfa (secondary outcome). This finding about C.E.R.A. is concordant with previous observations [23]. Retrospectively, we can suggest that the hemoglobin overshooting observed under C.E.R.A in the Q2W administration interval is probably due to an inadequate conversion factor (1:1) of ESAs dose in the transition from Q4W to Q2W administration interval (however, this was the conversion factor suggested by previous literature recommendations [9]) and to a too generous ESAs adjustment protocol in the maintenance phase (however approved by current labeling when the study was designed [11]).

The great sensibility of reticulocyte dynamic to ESAs therapy was evidenced by several factors, namely the evident oscillatory response induced by the ESAs pulse, the contracted short-term (week-to-week) variability when shortening the administration interval and splitting the ESAs dose, the amplified long-term (month-to-month) time instability under 2 week administration interval. Accordingly to its extended half-life, C.E.R.A. induced a more sustained erythropoietic response, as evidenced by the more protracted reticulocyte production under monthly administration intervals (secondary outcome). This can also explain the increased risk of hemoglobin overshooting (Hb > 12.0 g/dl) observed under C.E.R.A (secondary outcome).

When compared to reticulocytes, the hemoglobin dynamic seemed to be less sensitive to ESAs therapy strategy. No significant difference was found in the oscillatory course using the two different ESAs. The administration interval impacted on the monthly Hb course (loss of the cyclic oscillatory movement under Q2W administration), without modifying short-term week-to-week variability. The long-term (month-to-month) variability increased when shortening the administration interval from 4 to 2 weeks, and this change was predicted by the increased reticulocyte long-term (month-to-month) variability (primary outcome).

These results however, should be looked at carefully, taking into account the limitations of the study and the possible sources of bias.

First, in absence of previous literature studies quantifying the impact of different ESAs treatment and administration protocols on hemoglobin temporal instability in terms of both variability and temporal dependency over time, we were not able to assume a degree of effectiveness of therapy allowing us to estimate the sample size. We therefore proposed the protocol to all patients under hemodialysis at our centre at study begin.

Second, this is a randomized controlled study realized in a “real life” scenario. In order to assess the pharmacodynamic effect of ESAs on hemoglobin variability, an “on-treatment” analysis was performed, restricting the comparison of the treatments to the ideal patients-weeks, that is, those without intercurrent potentially perturbing event (hospitalization, transfusions, bleeding).

Ideally, the four administration protocols should have been studied in a stable population, without intercurrent illness, but this is far away of being the reality of a hemodialysis station. A further characteristic of the dialysis population is its high instability (deaths, transplants, dialysis centre change), that explains the high drop-out rate (only 50% of the patients included reached the study end).

## Conclusion

In conclusion, reticulocytes seemed to be a more sensitive marker of time instability of the erythropoietic dynamic under ESAs therapy, their variability influencing the hemoglobin response. As expected, shortening the administration interval lessened the amplitude of reticulocyte count fluctuations. However, surprisingly enough, the same strategy was associated with an increased month-to-month reticulocyte and hemoglobin instability. This meaning, that in the clinical setting the favorable and unfavorable consequences of lengthening the administration interval on hemoglobin variability tend to counterbalance each other, contrarily to the results of our previous published pharmacokinetic computer-based simulation. However, even if these results are comforting, the non-physiologic and extreme reticulocyte count fluctuation over time using the monthly administration interval imposes prudence and suggests further exploring the possible unfavorable consequences on hemoglobin stability. Furthermore, the more protracted reticulocyte response induced by C.E.R.A. (and even if the differences comparing to Darbepoetin alfa were less than expected) could explain both, the observed higher risk of hemoglobin overshoot and the significant increase in efficacy when shortening from once to twice a month its administration interval.

## Abbreviations

C.E.R.A: Continuous erythropoietin receptor activator; EPO: Erythropoietin; ESAs: Erythropoiesis stimulating agents; HD: Hemodialysis; Hb: Hemoglobin; Ret: Reticulocytes; ST-MSSD: Short-term mean square successive difference; LT-MSSD: Long-term mean square successive difference; Q4W: 4 Weeks interval; Q2W: 2 Weeks interval.

## Competing interests

The authors declare that they have no competing interests.

## Authors’ contributions

VF and LG were involved in the study design, analysis and interpretation of the data and writing of the report; GB and IS participated in the sample collection, and interpretation of the data; AO and PV participated to the data analysis. MB helped formulate the study design, the data analysis strategy and contributed to the writing of the paper. All authors read and approved the final manuscript.

## Pre-publication history

The pre-publication history for this paper can be accessed here:

http://www.biomedcentral.com/1471-2369/14/157/prepub
